# Problematic Smartphone and Social Media Use Among Bangladeshi College and University Students Amid COVID-19: The Role of Psychological Well-Being and Pandemic Related Factors

**DOI:** 10.3389/fpsyt.2021.647386

**Published:** 2021-04-16

**Authors:** Md. Saiful Islam, Md. Safaet Hossain Sujan, Rafia Tasnim, Rashenda Aziz Mohona, Most. Zannatul Ferdous, Sk Kamruzzaman, Tanziha Yeasmin Toma, Md. Nazmus Sakib, Khairrun Nahar Pinky, Md. Riad Islam, Md. Abid Bin Siddique, Fahim Shariar Anter, Alomgir Hossain, Ikram Hossen, Md. Tajuddin Sikder, Halley M. Pontes

**Affiliations:** ^1^Department of Public Health and Informatics, Jahangirnagar University, Savar, Bangladesh; ^2^Centre for Advanced Research Excellence in Public Health, Dhaka, Bangladesh; ^3^Department of Public Health, North South University, Dhaka, Bangladesh; ^4^Faculty of Animal Science and Veterinary Medicine, Patuakhali Science and Technology University, Barishal, Bangladesh; ^5^Department of Ayurvedic Medicine, Hamdard University Bangladesh, Gazaria, Bangladesh; ^6^Department of Population Science and Human Resource Development, Rajshahi University, Rajshahi, Bangladesh; ^7^Department of Anthropology, Rajshahi University, Rajshahi, Bangladesh; ^8^Department of Biochemistry and Molecular Biology, Noakhali Science and Technology University, Noakhali, Bangladesh; ^9^Department of Genetic Engineering and Biotechnology, Rajshahi University, Rajshahi, Bangladesh; ^10^Department of Organizational Psychology, Birkbeck, University of London, London, United Kingdom

**Keywords:** problematic use, smartphone, social media, college, university, students, Bangladesh

## Abstract

**Background:** Smartphone and social media use are an integral part of our daily life. Currently, the impact of excessive smartphone and social media use during the COVID-19 pandemic is poorly understood. The present study aimed to investigate problematic smartphone use (PSPU) and problematic social media use (PSMU) among Bangladeshi college and university students during the COVID-19 pandemic.

**Methods:** A cross-sectional study was carried out involving 5,511 Bangladeshi college and university students (male: 58.9%; mean age: 21.2 years [*SD* = 1.7]; age range: 18–25) during the social-distancing in the COVID-19 pandemic (July 2020). A self-reported survey containing questions regarding socio-demographic, lifestyle, and home quarantine activities along with four psychometric scales was completed by participants.

**Results:** The mean scores of PSPU and PSMU were 20.8 ± 6.8 (out of 36) and 14.7 ± 4.8 (out of 30). Based on a hierarchical regression analysis, PSPU and PSMU were positively associated with lower age, poor sleep, social media use, watching television, anxiety, and depression. Additionally, PSMU was linked to being female, living with nuclear family, having urban residence, irregular physical exercise, poor engagement with academic studies, and avoiding earning activities, whilst being male, being married, living with lower-income family, and alcohol consumption were linked to PSMU.

**Conclusions:** The findings indicate that PSPU and PSMU were linked to poor psychological well-being (i.e., anxiety and depression) and other factors (especially lower age, poor sleep) during the pandemic, further suggesting the need for interventions including virtual awareness programs among college and university students.

## Introduction

Currently, the world is facing a public health emergency declared by the World Health Organization due to international concerns regarding the highly contagious nature of COVID-19 (1). In Bangladesh (where the present study was carried out), the first three cases of COVID-19 were identified on March 8th 2020 (2, 3). Since then, the virus has continued to spread swiftly, and resulted in 430,496 positive cases with 6,173 fatalities by November 14th, 2020 (4, 5). As an immediate response to control the rapid spread of COVID-19, the Bangladesh government declared a “lock-down” or “stay at home order” on March 26, 2020 (6, 7). Everything including transports, shopping malls, offices, industries, and educational institutions got closed (8) and therefore, the government encouraged individuals to carry study, shopping, and work online at home. The initial restrictions (i.e., lock-down and social-distancing) aimed at controlling the spread of COVID-19 may have led to negative psychological impacts (e.g., anxiety, depression, frustration, fear, stress) to many individuals (9, 10). To help to cope with these negative mood states, individuals may engage in psychoactive substance use or engage problematically in specific technology use behaviors such as smartphone use, social media use, and video gaming as these potentially addictive behaviors may help alleviate the burden of stress and difficult thoughts experienced by individuals (11). However, the use of social media grew exponentially in the context of the COVID-19 pandemic (12). Higher amounts of time spent using social media may be problematic as these online platforms can be addictive (13). Social media platforms promote constant scrolling and do not have a definite “stop point,” which is why they can lead to individuals spending several hours using these online platforms (14). The use of social media is widespread amongst adolescents attending college and university (15–17). Recently, adolescents' smartphone use has been reported to constitute a potentially problematic issue (18, 19) due to the rise in smartphone use and its increased popularity through applications such as social media, gaming, streaming, and education to individuals with low levels of health literacy.

As shown by previous research, dysregulated use of smartphone is associated with sleep disturbance, and depressive symptoms among adolescents (20). The use of social media in classroom for educational purposes is becoming increasingly popular, with educators massively adopting it during the social-isolation period because of its enhanced communication potential, content distribution, collaborations, and engagement with the students (12). Furthermore, a recent study found that internet-based smartphone use can increase perceived quality of life by providing positive connections on social media, online shopping, online conferencing, and constant interaction with friends and family living in different countries (21).

Psychological health issues such as depression and anxiety have been positively linked with problematic smartphone use (PSPU) (22). Moreover, the COVID-19 pandemic and subsequent home quarantine and social-distancing requirements, have increased anxiety levels, and the extent of negative emotions experienced across society (23, 24), and through easily available means, many individuals seek emotional comfort by using smartphones and the internet as potential coping mechanisms. However, the adoption of such coping mechanisms may lead to many potential negative consequences because of functional impairments due to excessive usage (25, 26). PSPU has been characterized as severe levels of dysregulated smartphone use leading to problems in areas such as social, professional, and/or academic, with symptoms arguably similar to substance use disorders [e.g., withdrawal use, tolerance, careless use; (27)].

Bangladesh (where the present study was carried out) is a developing nation that is trying to become increasingly digitalized by enhancing internet use within the population, thus potentially increasing the extent of excessive usage of digital devices and the internet. At the end of January 2020, the total number of internet users in the country was over 99 million (28), and with this figure gradually increasing. Moreover, most of the country's internet users have smartphones. It is likely that, the required social-distancing measures may exacerbate excessive usage of smartphones and social media websites, especially among youth (29, 30).

To date, there are no previous studies focusing on PSPU and problematic social media use (PSMU) among college and university students in Bangladeshi in the context of the COVID-19 pandemic. Therefore, the present study aims to investigate the correlates of PSPU and PSMU with psychological well-being, socio-demographics, lifestyle, and home quarantine regular/frequent activities among college and university students in Bangladeshi during the COVID-19 pandemic. The study will inform future similar research and the development of new policies aiming to ascertain how Bangladeshi college and university students might be affected smartphone and social media use during the COVID-19 pandemic.

## Materials and Methods

### Participants and Procedure

The present cross-sectional study was conducted among Bangladeshi college and university students during the social-distancing period in the context of the COVID-19 pandemic. Data collection took place during in July 2020 using an online self-reported survey. All questions related to the survey were administered using Google Forms and a shareable link was generated to help disseminate the survey across different online platforms used by college and university students. The online survey detailed the study's informed consent, purpose, inclusion, and exclusion criteria on the first page. The inclusion criteria included (i) being 18 years old and above, (ii) being a Bangladeshi college or university student, (iii) owning a smartphone and having access to the internet, and (iv) being surveyed voluntarily. The exclusion criteria were (i) being under 18 years and (ii) not being completing the online survey entirely.

### Measures

A self-reported and structured online survey containing questions regarding participants' socio-demographic, lifestyle, and home quarantine activities along with four psychometric tests to measure the variables of the study were employed to collect data.

#### Socio-Demographic and Lifestyle Measures

Socio-demographic information concerning gender, age, marital status, education level, family type, and residence (urban/rural) was collected from all participants. Socio-economic status (SES) was categorized into three classes according to previous literature: lower, middle, and upper based on monthly family income of <15,000 Bangladeshi Taka (BDT) ≈ 177 US$, 15,000–30,000 BDT ≈ 177–354 US$, and more than 30,000 BDT ≈354 US$, respectively (31, 32). Moreover, numbers of average sleep hours classed as normal [7–9 h], less than normal [<7 h], or more than normal [>9 h] based on previous literature (33–35), physical exercising (yes/no), smoking cigarettes, and alcohol consumption (yes/no) were collected with regard to lifestyle measures.

#### Home Quarantine Activities Measures During COVID-19

Additional dichotomous questions (yes/no) were asked regarding the engagement in frequent activities during the pandemic, including home quarantine regular/frequent activities (i.e., academic/other studies, social-media use, watching television, household chores, and professional activities).

#### Patient Health Questionnaire (PHQ-9)

The PHQ−9 is a nine-item scale that measures depressive symptoms in the last 2 weeks (36). The items are rated from 0 (*Not at all*) to 3 (*Nearly every day*). Total scores can be obtained by summing up all responses on each item, and they range from 0 to 27. The present study used the Bangla version PHQ-9 (37) to assess participants' depressive symptoms as in previous research (38–40). The cutoff score ≥10 was used as a potential indication of depression in the sample recruited as previously in Bangladesh (32, 38, 41). In the present study, the PHQ-9 scale presented excellent internal consistency (Cronbach's alpha = 0.89).

#### Generalized Anxiety Disorder (GAD-7)

The GAD-7 is commonly used to screen symptoms and severity of generalized anxiety in epidemiological research (42). This scale consists of seven items that can be responded to on a four-point Likert scale ranging from 0 (*Not at all*) to 3 (*Nearly every day*). The present study adopted the Bangla version of the GAD-7 to assess participants' levels of anxiety as in previous research (39, 43, 44). The cutoff score ≥10 was used as indicative of anxiety as previously done in Bangladesh (39, 43). In the present study, the GAD-7 scale was observed as excellent reliability (Cronbach's alpha = 0.91).

#### Bergen Social Media Addiction Scale (BSMAS)

The BSMAS assesses PSMU with six questions based on the components model of addiction (i.e., salience, mood, modification, tolerance, withdrawal conflict, and relapse) proposed by Griffiths (49). The BSMAS was developed by Andreassen et al. (45), and was used to assess social media addiction by symptoms and related negative outcomes due to problematic use over the past year (e.g., “*How often during the last week have spent a lot of time thinking about social media or planned use of social media?*”). All items of the BSMAS can be responded using a five-point Likert scale ranging from 1 (*very rarely*) to 5 (*very often*). The total score is yielded by summating each items' raw score, with higher scores suggesting greater levels of PSMU symptoms. In the present study, the translated Bangla version of BSMAS was used to investigate PSMU. The BSMAS used in the study was adapted using a “back translation” procedure [i.e., (46)], similar to previous research conducted in Bangladesh (7, 47). In the present study, the Cronbach's alpha of BSMAS was 0.80.

#### Smartphone Application Based Addiction Scale (SABAS)

The SABAS was developed by Csibi et al. (48) and it consists of six items that measure PSPU. The scale was used to determine the extent of PSPU. The SABAS was developed based on the components model of addiction (i.e., salience, alteration of the mood, tolerance, withdrawal conflict, and relapse) which was proposed by Griffiths (49). Sample items include: “*During the past week, my smartphone is the most important thing in my life*.” All items are rated on a six-point Likert scale ranging from 1 (*strongly disagree*) to 6 (*strongly agree*). Total scores are summed up to reflect participants' overall levels of PSPU, with elevated scores suggesting a higher degree of PSPU. In the present study, the Bangla version of the SABAS was translated using a “back translation” procedure [i.e., (46)], similar to previous studies in Bangladesh (7, 47). In the present study, the Cronbach's alpha of the SABAS was 0.85.

### Statistical Analysis

Data were analyzed using Microsoft Excel 2019 and SPSS version 25. Firstly, the data set was cleaned, sorted, and coded using Microsoft Excel and exported onto SPSS. Using SPSS, descriptive statistics (i.e., frequencies, percentages, means, standard deviation estimates) were computed. Inferential statistics included conducting *t*-tests or one-way Analysis of Variance (ANOVA) to determine mean differences in the scores of the SABAS and BSMAS in relation to all variables examined applying Bonferroni correction (via dividing *p-*value significance threshold [0.05] into the number of independent variables: 0.05/18 = 0.003). Finally, multiple linear regression analyses predicting PSPU and PSMU across two independent models were conducted using a hierarchical approach including four blocks of predictors pertaining to: socio-demographic, lifestyle, home quarantine regular/frequent activities, and psychological well-being factors.

### Ethics

All procedures of the present study were performed in compliance with the Declaration of Helsinki, and adopting with guidelines of institutional research ethics. The present study has obtained ethical approval, which was given by the Ethical Review Committee (Ref. no: UAMC/ERC/17/2020). After being informed about the study's aims and procedures, all participants consented to participate in the study. Participants' information was strictly kept anonymous and confidential.

## Results

### Participants' Socio-Demographic Features

A total of 5,511 participants were included in the final analysis (see Table 1). Of these, 58.9% were male participants. The sample's average age was 21.2 years (*SD* = 1.7 years) and ages ranged from 18 to 25 years. The sample comprised college (22.9%) and university (77.1%) students. In terms of marital status, most participants were unmarried (95.8%). The majority of participants lived with their nuclear families (78.3%), held middle-class socioeconomic status (42.8%), and were from urban areas (50.5%). A sizeable minority did not engage in regular exercising (41.5%), and the majority were classed as normal sleepers (7–9 h/day) (67.6%). Last but not least, about 11.1% of all participants reported smoking cigarettes while a minority reported consuming alcohol (3.2%).

**Table 1:** Descriptive analyses of all examined variables (*N* = 5,511).

**Variables**	***n***	**(%)**
**SOCIO-DEMOGRAPHICS**		
**Gender**		
Male	3245	(58.9)
Female	2266	(41.1)
**Educational level**		
College	1260	(22.9)
University	4251	(77.1)
**Marital status**		
Unmarried	5281	(95.8)
Married	230	(4.2)
**Family type**		
Nuclear	4313	(78.3)
Joint	1198	(21.7)
**Socioeconomic status (SES)**		
Lower	1224	(22.2)
Middle	2358	(42.8)
Upper	1929	(35.0)
**Residence**		
Rural	2729	(49.5)
Urban	2782	(50.5)
**LIFESTYLE FACTORS**		
**Physical exercise**		
Yes	3224	(58.5)
No	2287	(41.5)
**Sleeping hours**		
Less than normal	1224	(22.2)
Normal (7–9 h)	3723	(67.6)
More than normal	564	(10.2)
**Smoking cigarettes**		
Yes	614	(11.1)
No	4897	(88.9)
**Alcohol consumption**		
Yes	179	(3.2)
No	5332	(96.8)
**HOME QUARANTINE REGULAR/FREQUENT ACTIVITIES**		
**Academic/other studies**		
Yes	4044	(73.4)
No	1467	(26.6)
**Social media use**		
Yes	5069	(92.0)
No	442	(8.0)
**Watching television**		
Yes	4723	(85.7)
No	788	(14.3)
**Household chores**		
Yes	4295	(77.9)
No	1216	(22.1)
**Earning activities**		
Yes	1523	(27.6)
No	3988	(72.4)
**PSYCHOLOGICAL WELL-BEING**		
**Anxiety**		
No	4027	(73.1)
Yes^a^	1484	(26.9)
**Depression**		
No	3416	(62.0)
Yes^b^	2095	(38.0)
**Continuous variables**	**Mean**	**(SD)**
Age	21.2	(1.8)
GAD-7	6.2	(5.74)
PHQ-9	6.2	(5.7)
SABAS	20.8	(6.8)
BSMAS	14.7	(4.8)

*GAD-7, Total score of Generalized Anxiety Disorder scale; PHQ-9, Total score of Patient Health Questionnaire scale; SABAS, Smartphone Application Based Addiction Scale; BSMAS, Bergen Social Media Addiction Scale*.

a *PHQ-9 ≥10*.

b *GAD-7 ≥10*.

### Associations Between PSPU and PSMU With Other Variables

The mean score of PSPU was 20.8 (*SD* = 6.8) out of 36. Moreover, high PSPU scores were positively associated with lower age, being female, being a university student, living in a nuclear family, being from an upper-class family, having urban residence, exhibiting poor physical exercising habits, greater sleep, smoking cigarettes, not consuming alcohol, not engaging with study, using social media, watching television shows, avoiding household chores, not in engaging earning activities, high levels of anxiety, and depression (Table 2).

**Table 2:** Associations between Problematic Smartphone Use (PSPU) and Problematic Social Media Use (PSMU) with all variables examined (*N* = 5,511).

**Variables**	***PSPU***				***PSMU***			
	**Mean**	**(SD)**	***t/F***	***p*-value**	**Mean**	**(SD)**	***t/F***	***p*-value**
**SOCIO-DEMOGRAPHICS**								
**Age**			1.92	0.002			3.09	<0.001
**Gender**								
Male	20.2	(7.0)	7.38	<0.001	14.6	(4.9)	4.00	<0.001
Female	21.8	(6.4)			14.8	(4.8)		
**Educational level**								
College	20.8	(6.9)	2.14	<0.001	15.2	(5.1)	3.61	<0.001
University	20.9	(6.8)			14.5	(4.8)		
**Relationship status**								
Unmarried	20.8	(6.8)	1.04	0.413	14.6	(4.8)	1.76	0.012
Married	20.8	(6.8)			15.3	(5.1)		
**Family type**								
Nuclear	21.2	(6.6)	14.25	<0.001	14.7	(4.8)	7.20	<0.001
Joint	19.5	(7.3)			14.4	(4.9)		
**Socioeconomic status (SES)**								
Lower	21.1	(6.2)	3.87	<0.001	15.2	(4.9)	4.42	<0.001
Middle	19.8	(7.2)			14.3	(4.8)		
Upper	21.9	(6.5)			14.8	(4.8)		
**Residence**								
Rural	19.9	(7.1)	9.40	<0.001	14.4	(4.9)	6.98	<0.001
Urban	21.8	(6.3)			15.0	(4.8)		
**LIFESTYLE FACTORS**								
**Physical exercise**								
Yes	19.5	(7.0)	14.31	<0.001	14.3	(4.9)	6.21	<0.001
No	22.7	(6.0)			15.2	(4.7)		
**Sleeping hours**								
Less than normal	21.5	(6.7)	2.41	<0.001	15.3	(4.9)	2.23	0.001
Normal (7–9 h)	20.4	(6.8)			14.4	(4.8)		
More than normal	22.0	(7.0)			15.3	(5.1)		
**Smoking habits**								
Yes	21.5	(6.7)	2.13	<0.001	15.2	(4.8)	2.10	0.001
No	20.8	(6.8)			14.6	(4.9)		
**Alcohol consumption**								
Yes	20.0	(7.3)	3.51	<0.001	15.6	(4.8)	3.33	<0.001
No	20.9	(6.8)			14.6	(4.8)		
**HOME QUARANTINE REGULAR/FREQUENT ACTIVITIES**								
**Academic/other studies**								
Yes	20.0	(6.9)	10.72	<0.001	14.4	(4.8)	5.08	<0.001
No	23.2	(6.1)			15.5	(4.8)		
**Social media use**								
Yes	21.3	(6.6)	33.13	<0.001	14.9	(4.8)	15.81	<0.001
No	15.8	(6.6)			12.5	(4.4)		
**Watching television**								
Yes	21.1	(6.8)	2.78	<0.001	14.7	(4.8)	2.57	<0.001
No	19.5	(7.0)			14.2	(4.9)		
**Household chores**								
Yes	20.5	(6.9)	6.65	<0.001	14.6	(4.8)	5.35	<0.001
No	22.0	(6.4)			15.0	(4.9)		
**Earning activities**								
Yes	18.0	(7.5)	27.01	<0.001	14.0	(5.1)	9.79	<0.001
No	21.9	(6.2)			14.9	(4.7)		
**PSYCHOLOGICAL WELL-BEING**								
**Anxiety**								
No	19.4	(6.6)	27.96	<0.001	13.5	(4.4)	47.82	<0.001
Yes	24.7	(5.7)			17.8	(4.5)		
**Depression**								
No	18.5	(6.5)	48.73	<0.001	12.9	(4.2)	69.69	<0.001
Yes	24.6	(5.5)			17.5	(4.4)		

The mean score of PSMU was 14.7 (*SD* = 4.8) out of 30. Similarly, high PSMU scores were positively associated with lower age, being female, being a college student, living in a nuclear family, being from a lower-class family, having urban residence, exhibiting poor physical exercising habits, less/more sleep, smoking cigarettes, alcohol consumption, not engaging with study, using social media, watching television, avoiding household chores, not engaging earning activities, high levels of anxiety, and depression (Table 2). Finally, a statistically significant positive correlation emerged between PSPU and PSMU (*r* = 0.61, *p* < 0.001).

### Factors Affecting PSPU and PSMU

The results of the hierarchical regression analysis predicting PSPU are presented in Table 3. Factors that were statistically significant in the group difference analyses (*t*-tests and ANOVA) were included in a hierarchical regression analysis. Socio-demographic factors (i.e., age, gender, educational level, family type, monthly family income, and residence) were included in Block 1. Lifestyle factors (i.e., physical exercise, sleeping hours, smoking cigarettes, and alcohol consumption) comprised Block 2. In Block 3, home quarantine activities (i.e., study, social media use, watching television, household chores, and earning activities) were included, while Block 4 comprised psychological well-being factors (i.e., anxiety and depression).

**Table 3:** Hierarchical regression analysis predicting Problematic Smartphone Use (PSPU).

**Model**	**Model 1**				**Model 2**				**Model 3**				**Model 4**					
	**B**	**SE**	**β**	***t***	**B**	**SE**	**β**	***t***	**B**	**SE**	**β**	***t***	**B**	**SE**	**β**	***t***	**ΔR^**2**^**	${\text{R}}_{\text{Adj}}^{2}$
**Block 1—Socio-demographics [*****F***_**(6, 5, 504)**_ **=** **33.74;** ***p*** **<** **0.001]**																	0.04	0.04
Age	−0.21	0.06	−0.05	−3.70^*^	−0.20	0.06	−0.05	−3.60^*^	−0.10	0.05	−0.03	−1.80	−0.10	0.05	−0.03	−2.03		
Gender^a^	1.12	0.19	0.08	5.87^*^	1.03	0.19	0.08	5.36^*^	0.83	0.19	0.06	4.42^*^	0.49	0.17	0.04	2.83		
Educational level^b^	0.43	0.23	0.03	1.83	0.33	0.23	0.02	1.45	−0.02	0.22	0.00	−0.11	0.34	0.20	0.02	1.70		
Family type^c^	−1.33	0.22	−0.08	−5.98^*^	−1.16	0.22	−0.07	−5.34^*^	−0.51	0.21	−0.03	−2.45	−0.51	0.19	−0.03	−2.68		
Socioeconomic status (SES)^d^	0.11	0.13	0.01	0.84	0.16	0.13	0.02	1.29	0.07	0.12	0.01	0.57	0.19	0.11	0.02	1.69		
Residence^e^	1.39	0.20	0.10	7.00^*^	1.11	0.19	0.08	5.73^*^	0.69	0.19	0.05	3.68^*^	0.34	0.17	0.03	2.00		
**Block 2—Lifestyle factors [*****F***_**(10, 5, 500)**_ **=** **50.21;** ***p*** **<** **0.001]**																	0.06	0.08
Physical exercise^f^					2.91	0.18	0.21	16.10^*^	1.84	0.18	0.13	9.99^*^	1.49	0.17	0.11	8.80^*^		
Sleeping hours^g^					−0.21	0.16	−0.02	−1.32	−0.33	0.15	−0.03	−2.20	−0.38	0.14	−0.03	−2.69		
Smoking habits^f^					−1.44	0.31	−0.07	−4.64^*^	−0.74	0.30	−0.03	−2.49	−0.13	0.27	−0.01	−0.48		
Alcohol consumption^f^					0.91	0.53	0.02	1.71	0.58	0.51	0.02	1.14	0.37	0.47	0.01	0.79		
**Block 3—Home quarantine regular/frequent activities [*****F***_**(15, 5, 495)**_ **=** **72.62;** ***p*** **<** **0.001]**																	0.14	0.16
Study^f^									2.08	0.20	0.14	10.40^*^	1.21	0.19	0.08	6.51^*^		
Social media use^f^									−4.13	0.32	−0.17	−12.91^*^	−3.55	0.29	−0.14	−12.10^*^		
Watching television^f^									−1.32	0.25	−0.07	−5.37^*^	−1.43	0.23	−0.07	−6.37^*^		
Household chores^f^									0.45	0.21	0.03	2.12	0.21	0.20	0.01	1.06		
Earning activities^f^									2.42	0.21	0.16	11.83^*^	2.16	0.19	0.14	11.53^*^		
**Block 4—Psychological well-being [*****F***_**(17, 5, 493)**_ **=** **139.12;** ***p*** **<** **0.001]**																	0.12	0.30
Anxiety^h^													1.65	0.22	0.11	7.49^*^		
Depression^h^													4.30	0.20	0.31	21.03^*^		

*B, unstandardized regression coefficient; SE, standard error; β, standardized regression coefficient*.

a *1 = Male, 2 = Female*.

b *1 = College, 2 = University*.

c *1 = Nuclear, 2 = Joint*.

d *1 = Lower, 2 = Middle, 3 = Upper*.

e *1 = Rural, 2 = Urban*.

f *1 = Yes, 2 = No*.

g *1 = <7 h, 2 = 7–9 h, 3 = >9 h*.

h *1 = Negative, 2 = Positive*.

* *p < 0.001*.

Overall, the regression model (Model 4) estimated predicted about 30% of the total variance in PSPU [*F*_(17, 5, 493)_= 139.12, *p* < 0.001]. In terms of specific predictors (applying Bonferroni correction), PSPU was predicted by irregular physical exercise, poor engagement with academic studies, social media use, watching television, avoiding earning activities, high levels of anxiety and depression. Consequently, age, gender, educational level, family type, monthly family income, residence, sleeping hours, smoking cigarettes, alcohol consumption, and household chores were not statistically significant predictors in the hierarchical regression analysis.

In relation to PSMU, the results of the hierarchical regression analysis indicated that the regression model (Model 4) predicted about 25% of the total variance in PSMU [*F*_(17, 5, 493)_= 110.44, *p* < 0.001; see Table 4]. Factors that were statistically significant in the group difference analyses (*t*-tests and ANOVA) were included in a hierarchical regression analysis. Socio-demographic factors (i.e., age, gender, educational level, family type, monthly family income, and residence) were included in Block 1. Lifestyle factors (i.e., physical exercise, sleeping hours, smoking cigarettes, and alcohol consumption) comprised Block 2. In Block 3, home quarantine activities (i.e., study, social media use, watching television, household chores, and earning activities) were included, and Block 4 comprised psychological well-being factors (i.e., anxiety and depression). Accordingly, PSMU was predicted (applying Bonferroni correction) by younger age, poor sleep (<7 h/day), alcohol consumption, social media use, high levels of anxiety, and depression. Consequently, gender, educational level, family type, monthly family income, residence, physical exercise, smoking cigarettes, study, watching television, household chores, and earning activities were not statistically significant predictors in the hierarchical regression analysis.

**Table 4:** Hierarchical regression analysis predicting Problematic Social Media Use (PSMU).

**Model**	**Model 1**				**Model 2**				**Model 3**				**Model 4**					
	**B**	**SE**	**β**	***t***	**B**	**SE**	**β**	***t***	**B**	**SE**	**β**	***t***	**B**	**SE**	**β**	***t***	**ΔR^**2**^**	${\text{R}}_{\text{Adj}}^{2}$
**Block 1—Socio-demographics [*****F***_**(6, 5, 504)**_ **=** **10.38;** ***p*** **<** **0.001]**																	0.01	0.01
Age	−0.14	0.04	−0.05	−3.55^**^	−0.15	0.04	−0.06	−3.75^**^	−0.13	0.04	−0.05	−3.18^*^	−0.13	0.04	−0.05	−3.61^**^		
Gender^a^	0.05	0.14	0.01	0.35	0.11	0.14	0.01	0.80	0.07	0.14	0.01	0.50	−0.24	0.13	−0.03	−1.91		
Educational level^b^	−0.42	0.17	−0.04	−2.51	−0.43	0.17	−0.04	−2.60	−0.56	0.17	−0.05	−3.37^*^	−0.25	0.15	−0.02	−1.74		
Family type^c^	−0.13	0.16	−0.01	−0.82	−0.07	0.16	−0.01	−0.45	0.20	0.16	0.02	1.25	0.20	0.14	0.02	1.38		
Socioeconomic status (SES)^d^	0.11	0.09	−0.04	−2.76	−0.24	0.09	−0.04	−2.53	−0.27	0.09	−0.04	−2.89	−0.16	0.08	−0.03	−1.99		
Residence^d^	0.71	0.14	0.07	4.96^**^	0.62	0.14	0.06	4.33^**^	0.50	0.14	0.05	3.53^**^	0.20	0.13	0.02	1.56		
**Block 2—Lifestyle factors [*****F***_**(10, 5, 500)**_ **=** **12.30;** ***p*** **<** **0.001]**																	0.01	0.02
Physical exercise^f^					0.83	0.13	0.08	6.20^**^	0.50	0.14	0.05	3.60^**^	0.20	0.12	0.02	1.59		
Sleeping hours^g^					−0.25	0.12	−0.03	−2.14	−0.29	0.12	−0.03	−2.54	−0.32	0.10	−0.04	−3.12^*^		
Smoking habits^f^					−0.66	0.23	−0.04	−2.87	−0.40	0.23	−0.03	−1.76	0.15	0.20	0.01	0.75		
Alcohol consumption^f^					−0.74	0.39	−0.03	−1.89	−0.87	0.39	−0.03	−2.24	−1.06	0.34	−0.04	−3.09^*^		
**Block 3—Home quarantine regular/frequent activities [*****F***_**(15, 5, 495)**_ **=** **17.81;** ***p*** **<** **0.001]**																	0.02	0.04
Study^f^									0.89	0.15	0.08	5.81^**^	0.12	0.14	0.01	0.90		
Social media use^f^									−2.22	0.24	−0.12	−9.11^**^	−1.70	0.22	−0.10	−7.89^**^		
Watching television^f^									−0.33	0.19	−0.02	−1.79	−0.46	0.17	−0.03	−2.77		
Household chores^f^									0.09	0.16	0.01	0.55	−0.12	0.14	−0.01	−0.80		
Earning activities^f^									0.36	0.16	0.03	2.29	0.14	0.14	0.01	0.99		
**Block 4—Psychological well-being [*****F***_**(17, 5, 493)**_ **=** **110.44;** ***p*** **<** **0.001]**																	0.21	0.25
Anxiety^h^													1.92	0.16	0.18	11.90^**^		
Depression^h^													3.44	0.15	0.34	22.88^**^		

*B, unstandardized regression coefficient; SE, standard error; β, standardized regression coefficient*.

a *1 = Male, 2 = Female*.

b *1 = College, 2 = University*.

c *1 = Nuclear, 2 = Joint*.

d *1 = Lower, 2 = Middle, 3 = Upper*.

e *1 = Rural, 2 = Urban*.

f *1 = Yes, 2 = No*.

g *1 = <7 h, 2 = 7–9 h, 3= >9 h*.

h *1 = Negative, 2 = Positive*.

* p < 0.003 and

** *p < 0.001*.

## Discussion

Since the beginning of the COVID-19 pandemic, the use of smartphone and social media has rapidly increased globally, with digital technology use being associated with poorer mental health (50). Throughout modern societies, smartphone use became pervasive and can be seen across the entire lifespan. Moreover, social media and smartphone use has become essential in daily life as it provides access to a wide range of mobile applications, information, communication, education, and entertainment tools. The use of smartphones and the internet are a cause for concern for various communities due to the deleterious effects stemming from problematic use (51, 52).

To the best of the authors' knowledge, this is the first study investigating the intricacies between PSPU and PSMU in relation to key associated factors during the COVID-19 pandemic in Bangladesh. According to the findings obtained in this study, PSMU was positively associated with irregular physical exercise, poor engagement with academic studies, social media use, watching television, ignoring earning activities, anxiety, and depression. Similarly, PSMU was positively associated with lower age, poor sleep, alcohol consumption, social media use, anxiety, and depression. Moreover, according to the hierarchical regression analyses conducted, individuals with irregular physical activity were found to exhibit higher levels of PSPU than physically active individuals, a finding that corroborates the results of previous research conducted in Korea (53) and Switzerland (54) reporting that problematic users were less willing to exercise regularly.

The present study found that PSPU was significantly associated with poor study engagement. Previous studies reported that PSPU has an adverse effect on academic performance (55, 56). This finding is aligned with existing evidence indicating that prolonged smartphone use in adolescents is linked with poorer academic performance (52). Additionally, students at high risk of PSPU are less likely to have high grade point averages (GPAs) (57). Furthermore, PSPU was higher among social media users, which is consistent with previous research indicating that PSMU can occur among smartphone users (58).

Using smartphone as a source of entertainment (e.g., social media), has been found to be linked with PSPU according to an earlier study (59). Furthermore, participants using social media through their smartphones have been found to spend 0.5–3 h daily using social media (60), with greater time spent using social media posing greater addictive risk, and potentially contributing to greater PSPU risk (58). Moreover, the results of this study found that depression, and anxiety were positively associated with PSPU, which is consistent with several preceding studies showing a significant association between PSPU and both depression and anxiety symptoms (59, 61, 62). According to the findings obtained in this study, lower age was found to be positively associated with PSMU, which is similar to an earlier study (63). During home quarantine and self-isolation period, the main way to meet new people and socialize was through social media platforms, which potentially contributing to greater time spent on social media sites in order to alleviate the venerable psychological sates brought about by the pandemic (64, 65). Social media in today's society is widespread and, with excessive usage potentially leading to PSMU, particularly young people. Although social media may be used to communicate and establish connections with friends and significant others, excessive use can occur, leading to the development of PSMU (66).

In the hierarchical regression analysis conducted, reporting with less sleep (<7 h/day) were more prone to PSMU. An earlier study in the same country also supports this finding (67). Research has shown that both inadequate and prolonged sleep (68) are associated with PSMU. Another important finding was that alcohol consumption and PSMU were positively associated in the present study. Participants consuming alcohol may become more engaged in alcohol-related social media usage (69), spending more time using social media, which may lead to excessive use. Depression and anxiety were also positively associated with PSMU according to the findings of obtained. Several preceding studies have found an association between both depression and anxiety with PSMU (61, 67, 70–72), with longitudinal research showing a strong association between depression and trend of internet use (73).

The present study investigated potential factors associated with PSPU and PSMU during the COVID-19 pandemic. We have examined several factors (i.e., psychological well-being, socio-demographics, lifestyle, and home quarantine activities) predicting PSPU and PSMU during home quarantine in the context of the COVID-19 pandemic. Further evaluation is needed after the pandemic in order to estimate the potential psychiatric toll in terms of post-traumatic stress disorder symptoms among both excessive and problematic digital technology users.

### Limitations

The present study is not without potential limitations. Firstly, causality cannot be established based on the findings reported due to the cross-sectional design adopted. As we did not have pre-COVID-19 data, the findings obtained cannot be causally inferred in that the COVID-19 pandemic leads to PSPU and PSMU. In this context, longitudinal and experimental studies will assist in exploring causal factors leading to PSPU and PSMU. Secondly, this study employed a self-report approach, which is likely to introduce specific biases (e.g., social desirability and memory recall biases). Furthermore, this study was conducted on a convenience sample of college and university students from Bangladesh, which cannot be representative of other populations and users around the globe. So, a longitudinal study focusing on different groups is needed.

## Conclusions

The present study focused on the factors associated with PSPU and PSMU during the COVID-19 pandemic. It can therefore be concluded that the COVID-19 pandemic led to an innovative utilization of smartphone and social media that helped keeping the population informed and socially connected during the COVID-19 pandemic, but excessive use may become an issue, leading to problematic usage. Psychosocial education and counseling programs should be implemented to prevent and mitigate the development of PSPU and PSMU and related psychological problems especially among vulnerable populations.

## Data Availability Statement

The raw data supporting the conclusions of this article will be made available by the authors, without undue reservation.

## Ethics Statement

The studies involving human participants were reviewed and approved by the Ethical Review Committee, Uttara Adhunik Medical College, Uttara, Dhaka-1260, Bangladesh (Ref. no: UAMC/ERC/17/2020). The patients/participants provided their written informed consent to participate in this study.

## Author Contributions

MSI, MSuj, RT, and MF: conceptualization. MSI, MSuj, and MF: methodology. MSI: formal analysis and investigation. MSI, MSuj, RT, and RM: writing—original draft preparation. MSI, MF, MSik, and HP: writing—review and editing. SK, TT, MSak, KP, MRI, MSid, FA, AH, and IH: resources. MF and MSik: supervision. All authors read and approved the final manuscript.

## Conflict of Interest

The authors declare that the research was conducted in the absence of any commercial or financial relationships that could be construed as a potential conflict of interest.

## References

[1] Mahase E. China coronavirus: WHO declares international emergency as death toll exceeds 200. BMJ. (2020) 368:m408. 10.1136/bmj.m408 32005727

[2] Ferdous MZ Islam MS Sikder MT Mosaddek ASM Zegarra-Valdivia JA Gozal D. Knowledge, attitude, and practice regarding COVID-19 outbreak in Bangladesh: an online-based cross-sectional study. PLoS ONE. (2020) 15:e0239254. 10.1371/journal.pone.0239254 33035219 PMC7546509

[3] Islam MS Emran GI Rahman E Banik R Sikder T Smith L. Knowledge, attitudes and practices associated with the COVID-19 among slum dwellers resided in Dhaka City: a Bangladeshi interview-based survey. J Public Health. (2020). 10.1093/pubmed/fdaa182. [Epub ahead of print]. 33057666 PMC7665690

[4] Institute of Epidemiology Disease Control and Research. Covid-19 Status for Bangladesh. (2020). Available online at: http://old.iedcr.gov.bd/ (accessed June 28, 2020).

[5] Worldometer. COVID-19 Coronavirus Pandemic. (2020). Available online at: https://www.worldometers.info/coronavirus (accessed July 21, 2020).

[6] Islam M Sujan SH Tasnim R Sikder T Potenza M Van Os J. Psychological responses during the COVID-19 outbreak among university students in Bangladesh. PLoS ONE. (2020) 15:e0245083. 10.31234/osf.io/cndz7 33382862 PMC7775049

[7] Tasnim R Sujan MSH Islam MS Ritu AH Siddique MA Bin Toma TY. Prevalence and correlates of anxiety and depression in frontline healthcare workers treating people with COVID-19 in Bangladesh. PsyArXiv. (2020). 10.31234/osf.io/3qg9p PMC8146174 34034679

[8] Islam MA Barna SD Raihan H Khan MNA Hossain MT. Depression and anxiety among university students during the COVID-19 pandemic in Bangladesh: a web-based cross-sectional survey. PLoS ONE. (2020) 15:e0238162. 10.1371/journal.pone.0238162 32845928 PMC7449469

[9] Banerjee D. The COVID-19 outbreak: crucial role the psychiatrists can play. Asian J Psychiatr. (2020) 50:102014. 10.1016/j.ajp.2020.102014 32240958 PMC7270773

[10] Islam MS Potenza MN Van Os J. Posttraumatic stress disorder during the COVID-19 pandemic: upcoming challenges in Bangladesh and preventive strategies. Int J Soc Psychiatry. (2020). 10.1177/0020764020954469. [Epub ahead of print]. 32873127

[11] Blasi M Di Giardina A Giordano C Coco G Lo Tosto C Billieux J. Problematic video game use as an emotional coping strategy: Evidence from a sample of MMORPG gamers. J Behav Addict J Behav Addict. (2019) 8:25–34. 10.1556/2006.8.2019.02 30739460 PMC7044601

[12] Gupta K DSilva MH. Proliferation of social media during the COVID-19 pandemic: a statistical enquiry. J Xi'an Univ Archit Technol. (2020) 12:1752–9.

[13] Alter A. Irresistible: The Rise of Addictive Technology and the Business of Keeping us Hooked. Penguin (2017).

[14] Garfin DR. Technology as a coping tool during the coronavirus disease 2019 (COVID-19) pandemic: implications and recommendations. Stress Heal J Int Soc Investig Stress. (2020) 36:555–9. 10.1002/smi.2975 32762116 PMC7436915

[15] Tess PA. The role of social media in higher education classes (real and virtual)—a literature review. Comput Human Behav. (2013) 29:A60–A68. 10.1016/j.chb.2012.12.032

[16] Andreassen CS Pallesen S Griffiths MD. The relationship between addictive use of social media, narcissism, and self-esteem: findings from a large national survey. Addict Behav. (2017) 64:287–93. 10.1016/j.addbeh.2016.03.006 27072491

[17] Bányai F Zsila Á Király O Maraz A Elekes Z Griffiths MD. Problematic social media use: results from a large-scale nationally representative adolescent sample. PLoS ONE. (2017) 12:e0169839. 10.1371/journal.pone.0169839 28068404 PMC5222338

[18] Kim D Lee Y Lee J Nam JK Chung Y. Development of Korean smartphone addiction proneness scale for youth. PLoS ONE. (2014) 9:e97920. 10.1371/journal.pone.0097920 24848006 PMC4029762

[19] Lee C Lee S-J. Prevalence and predictors of smartphone addiction proneness among Korean adolescents. Child Youth Serv Rev. (2017) 77:10–17. 10.1016/j.childyouth.2017.04.002

[20] Lemola S Perkinson-Gloor N Brand S Dewald-Kaufmann JF Grob A. Adolescents' electronic media use at night, sleep disturbance, and depressive symptoms in the smartphone age. J Youth Adolesc. (2015) 44:405–18. 10.1007/s10964-014-0176-x 25204836

[21] Pontes HM Szabo A Griffiths MD. The impact of Internet-based specific activities on the perceptions of internet addiction, quality of life, and excessive usage: a cross-sectional study. Addict Behav Rep. (2015) 1:19–25. 10.1016/j.abrep.2015.03.002 29531976 PMC5845921

[22] Yang J Fu X Liao X Li Y. Association of problematic smartphone use with poor sleep quality, depression, and anxiety: a systematic review and meta-analysis. Psychiatry Res. (2020) 284:112686. 10.1016/j.psychres.2019.112686 31757638

[23] Gao J Zheng P Jia Y Chen H Mao Y Chen S. Mental health problems and social media exposure during COVID-19 outbreak. PLoS ONE. (2020) 15:e0231924. 10.1371/journal.pone.0231924 32298385 PMC7162477

[24] Qiu J Shen B Zhao M Wang Z Xie B Xu Y. A nationwide survey of psychological distress among Chinese people in the COVID-19 epidemic: implications and policy recommendations. Gen Psychiatry. (2020) 33:e100213. 10.1136/gpsych-2020-100213 32215365 PMC7061893

[25] Elhai JD Yang H McKay D Asmundson GJG. COVID-19 anxiety symptoms associated with problematic smartphone use severity in Chinese adults. J Affect Disord. (2020) 274:576–582. 10.1016/j.jad.2020.05.080 32663990 PMC7251360

[26] Brand M Young KS Laier C Wölfling K Potenza MN. Integrating psychological and neurobiological considerations regarding the development and maintenance of specific Internet-use disorders: an Interaction of Person-Affect-Cognition-Execution (I-PACE) model. Neurosci Biobehav Rev. (2016) 71:252–66. 10.1016/j.neubiorev.2016.08.033 27590829

[27] Ryding FC Kuss DJ. Passive objective measures in the assessment of problematic smartphone use: a systematic review. Addict Behav Rep. (2020) 11:100257. 10.1016/j.abrep.2020.100257 32467846 PMC7244920

[28] Bangladesh Telecommunication Regulatory Commission. Internet Subscribers in Bangladesh January, 2020. (2020). Available online at: http://www.btrc.gov.bd/telco/internet (accessed March 21, 2020).

[29] Dong H Yang F Lu X Hao W. Internet addiction and related psychological factors among children and adolescents in china during the coronavirus disease 2019 (COVID-19) epidemic. Front Psychiatry. (2020) 11:751. 10.3389/fpsyt.2020.00751 32982806 PMC7492537

[30] Hamilton J Nesi J Choukas-Bradley S. Teens and social media during the COVID-19 pandemic: staying socially connected while physically distant. PsyArXiv. (2020). 10.31234/osf.io/5stx4

[31] Rahman ME Islam MS Bishwas MS Moonajilin MS Gozal D. Physical inactivity and sedentary behaviors in the Bangladeshi population during the COVID-19 pandemic: an online cross-sectional survey. Heliyon. (2020) 6:e05392. 10.1016/j.heliyon.2020.e05392 33163666 PMC7598079

[32] Rahman ME Islam MS Mamun MA Moonajilin MS Yi S. Prevalence and factors associated with suicidal ideation among university students in Bangladesh. Arch Suicide Res. (2020) 1–10. 10.1080/13811118.2020.1833800. [Epub ahead of print]. 33073746

[33] Islam MS Akter R Sikder MT Griffiths MD. Weight-related status and associated predictors with psychological well-being among first-year university students in Bangladesh: a pilot study. Int J Ment Health Addict. (2020). 10.1007/s11469-020-00243-x. [Epub ahead of print].

[34] Hirshkowitz M Whiton K Albert SM Alessi C Bruni O DonCarlos L. National Sleep Foundation's updated sleep duration recommendations: final report. Sleep Heal. (2015) 1:233–43. 10.1016/j.sleh.2015.10.004 29073398

[35] Islam MS Sujan MSH Tasnim R Ferdous MZ Masud JHB Kundu S. Problematic internet use among young and adult population in Bangladesh: correlates with lifestyle and online activities during the COVID-19 pandemic. Addict Behav Rep. (2020) 12:100311. 10.1016/j.abrep.2020.100311 33364319 PMC7752719

[36] Spitzer RL Kroenke K Williams JB. Validation and utility of a self-report version of PRIME-MD: the PHQ primary care study. Primary Care Evaluation of Mental Disorders. Patient Health Questionnaire. JAMA. (1999) 282:1737–44. 10.1001/jama.282.18.1737 10568646

[37] Chowdhury A Ghosh S Sanyal D. Bengali adaptation of Brief Patient Health Questionnaire for screening depression at primary care. J Indian Med Assoc. (2004) 102:544–7. 15887819

[38] Islam MS Akter R Sikder T Griffiths MD. Prevalence and factors associated with depression and anxiety among first-year university students in Bangladesh: a cross-sectional study. Int J Ment Health Addict. (2020). 10.1007/s11469-020-00242-y. [Epub ahead of print].

[39] Moonajilin MS Rahman ME Islam MS. Relationship between overweight/obesity and mental health disorders among Bangladeshi adolescents: a cross-sectional survey. Obes Med. (2020) 18:100216. 10.1016/j.obmed.2020.100216

[40] Islam MS Rahman ME Banik R Emran MGI Noshin S Hossain S. Correlates of financial concerns, depression symptoms, and posttraumatic stress disorder symptoms among impoverished urban dwelling individuals in Bangladesh during the COVID-19 pandemic: a face-to-face interview approach. PsyArXiv. (2020). 10.31234/osf.io/nfr5m

[41] Islam MS Ferdous MZ Islam US Mosaddek ASM Potenza MN Pardhan S. Treatment, persistent symptoms, and depression in people infected with COVID-19 in Bangladesh. Int J Environ Res Public Health. (2021) 18:1453. 10.3390/ijerph18041453 33562427 PMC7914967

[42] Spitzer RL Kroenke K Williams JBW Löwe B. A brief measure for assessing generalized anxiety disorder: the GAD-7. Arch Intern Med. (2006) 166:1092–7. 10.1001/archinte.166.10.1092 16717171

[43] Islam MS Ferdous MZ Potenza MN. Panic and generalized anxiety during the COVID-19 pandemic among Bangladeshi people: an online pilot survey early in the outbreak. J Affect Disord. (2020) 276:30–7. 10.1016/j.jad.2020.06.049 32697713 PMC7362838

[44] Haque M Das C Ara R Alam M Ullah S Hossain Z. Prevalence of generalized anxiety disorder and its effect on daily living in the rural community of Rajshahi. J Teach Assoc. (2018) 27:14–23. 10.3329/taj.v27i1.37603

[45] Andreassen CS Billieux J Griffiths MD Kuss DJ Demetrovics Z Mazzoni E. The relationship between addictive use of social media and video games and symptoms of psychiatric disorders: a large-scale cross-sectional study. Psychol Addict Behav. (2016) 30:252–62. 10.1037/adb0000160 26999354

[46] Beaton DE Bombardier C Guillemin F Ferraz MB. Guidelines for the process of cross-cultural adaptation of self-report measures. Spine. (2000) 25:3186–91. 10.1097/00007632-200012150-00014 11124735

[47] Islam MS Rahman ME Moonajilin MS Griffiths MD. Validation and evaluation of the psychometric properties of Bangla nine-item Internet Disorder Scale–Short Form. J Addict Dis. (2020) 38:540–9. 10.1080/10550887.2020.1799134 32762512

[48] Csibi S Griffiths MD Cook B Demetrovics Z Szabo A. The psychometric properties of the Smartphone Application-Based Addiction Scale (SABAS). Int J Ment Health Addict. (2018) 16:393–403. 10.1007/s11469-017-9787-2 29670500 PMC5897481

[49] Griffiths MA ‘components' model of addiction within a biopsychosocial framework. J Subst Use. (2005) 10:191–7. 10.1080/14659890500114359

[50] Zhao N Zhou G. Social media use and mental health during the COVID-19 pandemic: moderator role of disaster stressor and mediator role of negative affect. Appl Psychol Health Well Being. (2020) 12:1019–38. 10.1111/aphw.12226 32945123 PMC7536964

[51] Harris B Regan T Schueler J Fields SA. Problematic mobile phone and smartphone use scales: a systematic review. Front Psychol. (2020) 11:672. 10.3389/fpsyg.2020.00672 32431636 PMC7214716

[52] Liu X Luo Y Liu Z-Z Yang Y Liu J Jia C-X. Prolonged mobile phone use is associated with poor academic performance in adolescents. Cyberpsychol Behav Soc Netw. (2020) 23:303–11. 10.1089/cyber.2019.0591 32191529

[53] Kim S-E Kim J-W Jee Y-S. Relationship between smartphone addiction and physical activity in Chinese international students in Korea. J Behav Addict JBA. (2015) 4:200–5. 10.1556/2006.4.2015.028 26551911 PMC4627682

[54] Haug S Castro RP Kwon M Filler A Kowatsch T Schaub MP. Smartphone use and smartphone addiction among young people in Switzerland. J Behav Addict. (2015) 4:299–307. 10.1556/2006.4.2015.037 26690625 PMC4712764

[55] Chaudhury P Tripathy H. A Study on impact of smartphone addiction on academic performance. Int J Eng Technol. (2018) 7:50. 10.14419/ijet.v7i2.6.10066

[56] Cao X Masood A Luqman A Ali A. Excessive use of mobile social networking sites and poor academic performance: antecedents and consequences from stressor-strain-outcome perspective. Comput Human Behav. (2018) 85:163–74. 10.1016/j.chb.2018.03.023

[57] Hawi NS Samaha M. To excel or not to excel: strong evidence on the adverse effect of smartphone addiction on academic performance. Comput Educ. (2016) 98:81–9. 10.1016/j.compedu.2016.03.007

[58] Giunchiglia F Zeni M Gobbi E Bignotti E Bison I. Mobile social media usage and academic performance. Comput Human Behav. (2018) 82:177–85. 10.1016/j.chb.2017.12.041

[59] Matar Boumosleh J Jaalouk D. Depression, anxiety, and smartphone addiction in university students- A cross sectional study. PLoS ONE. (2017) 12:e0182239. 10.1371/journal.pone.0182239 28777828 PMC5544206

[60] Owusu-Acheaw M Larson AG. Use of social media and its impact on academic performance of tertiary institution students: a study of students of Koforidua Polytechnic, Ghana. J Educ Pract. (2015) 6:94–101.

[61] Alhassan AA Alqadhib EM Taha NW Alahmari RA Salam M Almutairi AF. The relationship between addiction to smartphone usage and depression among adults: a cross sectional study. BMC Psychiatry. (2018) 18:148. 10.1186/s12888-018-1745-4 29801442 PMC5970452

[62] Chen B Liu F Ding S Ying X Wang L Wen Y. Gender differences in factors associated with smartphone addiction: a cross-sectional study among medical college students. BMC Psychiatry. (2017) 17:341. 10.1186/s12888-017-1503-z 29017482 PMC5634822

[63] Blackwell D Leaman C Tramposch R Osborne C Liss M. Extraversion, neuroticism, attachment style and fear of missing out as predictors of social media use and addiction. Pers Individ Diff. (2017) 116:69–72. 10.1016/j.paid.2017.04.039

[64] Singh S Dixit A Joshi G. "Is compulsive social media use amid COVID-19 pandemic addictive behavior or coping mechanism? Asian J Psychiatr. (2020) 54:102290. 10.1016/j.ajp.2020.102290 32659658 PMC7338858

[65] Wiederhold BK. Using social media to our advantage: alleviating anxiety during a pandemic. Cyberpsychol Behav Soc Netw. (2020) 23:197–8. 10.1089/cyber.2020.29180.bkw 32207630

[66] Ramesh Masthi N Cadabam S Sonakshi S. Facebook addiction among health university students in Bengaluru. Int J Heal Allied Sci. (2015) 4:18–22. 10.4103/2278-344X.149234

[67] Mamun MA Al Griffiths MD. The association between Facebook addiction and depression: a pilot survey study among Bangladeshi students. Psychiatry Res. (2019) 271:628–33. 10.1016/j.psychres.2018.12.039 30791335

[68] Islam MA Hossin MZ. Prevalence and risk factors of problematic internet use and the associated psychological distress among graduate students of Bangladesh. Asian J Gambl Issues Public Heal. (2016) 6:11. 10.1186/s40405-016-0020-1 27942430 PMC5122610

[69] Curtis BL Lookatch SJ Ramo DE McKay JR Feinn RS Kranzler HR. Meta-analysis of the association of alcohol-related social media use with alcohol consumption and alcohol-related problems in adolescents and young adults. Alcohol Clin Exp Res. (2018) 42:978–86. 10.1111/acer.13642 29786874 PMC5984178

[70] Primack BA Shensa A Escobar-Viera CG Barrett EL Sidani JE Colditz JB. Use of multiple social media platforms and symptoms of depression and anxiety: a nationally-representative study among U.S. young adults. Comput Human Behav. (2017) 69:1–9. 10.1016/j.chb.2016.11.013 PMC13268370 42305949

[71] Shensa A Escobar-Viera CG Sidani JE Bowman ND Marshal MP Primack BA. Problematic social media use and depressive symptoms among U.S. young adults: a nationally-representative study. Soc Sci Med. (2017) 182:150–7. 10.1016/j.socscimed.2017.03.061 28446367 PMC5476225

[72] Pontes HM. Investigating the differential effects of social networking site addiction and Internet gaming disorder on psychological health. J Behav Addict. (2017) 6:601–10. 10.1556/2006.6.2017.075 29130329 PMC6034963

[73] Strong C Lee C-T Chao L-H Lin C-Y Tsai M-C. Adolescent Internet use, social integration, and depressive symptoms: analysis from a longitudinal cohort survey. J Dev Behav Pediatr. (2018) 39:318–24. 10.1097/DBP.0000000000000553 29461298

