# Supplementary material for: Problematic Smartphone and Social Media Use Among Bangladeshi College and University Students Amid COVID-19: The Role of Psychological Well-Being and Pandemic Related Factors
Source: Front Psychiatry. 2021 Apr 16;12:647386. doi: 10.3389/fpsyt.2021.647386 (PMC8085355; doi:10.3389/fpsyt.2021.647386)
Supplement: Supplementary file 1 [file Table_1.DOCX]

Acknowledgments: The volunteers who significantly contributed during the data collection periods

| Name | Institution | Email address |
| --- | --- | --- |
| Md Ashraful Amin | **Rajshahi University** | **asrafulamin43@gmail.com** |
| Abdul Kaium | **Rajshahi University** | **abdulkaium56765@gmail.com** |
| Md. Sariful Islam | **Jahangirnagar University** | **ridoynku143@gmail.com** |
| Taniza Tasnim | **Independent University, Bangladesh** | **tanizatasnim@gmail.com** |
| Ali Hossain Roni | **Patuakhali Science and Technology University** | **arhossain57@gmail.com** |
| Shahana Akhter Iren | **Rajshahi University** | **shahana1998iren@gmail.com** |
| Partha Sarathi Roy | **Noakhali Science and Technology University** | **parthasarathiroy1999@gmail.com** |
| Tanjina Akter Shamme | **Jagannath University** | **angelshammi78@gmail.com** |
| Adittya Kishor Chakraborty | **State College of Health Sciences** | **adittyakc777@gmail.com** |
| Nadia Mahzabin Muna | **Sheikh Hasina University** | **nadiamahzabin2000@gmail.com** |
| Md.Saiful Islam | **Begum Rokeya University** | **saifulislamsaif1357@gmail.com** |
| Jannatul Ferdous | **Patuakhali Science and Technology University** | **ferdousjannatul4769@gmail.com** |
| Raisul Islam | **Rajshahi University** | **raisul.islam2000@outlook.com** |
| Md. Raihan Khandaker | **Patuakhali Science and Technology University** | **raihaner77@gmail.com** |
| Md. Abdullah Al Mamun | **Cumilla University** | **mdabdullahalmamun932@gmail.com** |
| Mitu Datto | **Patuakhali Science and Technology University** | **dattomitu123@gmail.com** |
| Md.Farah Eydid | **Patuakhali Science and Technology University** | **farhansaad1601056@gmail.com** |
| Md Arif Ullah | **University of Chittagong** | **arifctg814@gmail.com** |
| Md Masukur Rahman Masuk | **Rajshahi University** | **mrmasuk25@gmail.com** |
| Md Amit Hasan | **Patuakhali Science and Technology University** | **hasanamit789@gmail.com** |
| Ireen Nahar | **Patuakhali Science and Technology University** | **ireen89eti@gmail.com** |
| Umme Tabassum Jahan Akand | **University of Rajshahi** | **tabassummira42@gmail.com** |
| Most. Sumi Khatun | **Sirajganj Govt. College** | **sumikhatun121273@gmail.com** |
| Shaikh Mansura Akter | **University of Rajshahi** | **mansura.rifa2000@gmail.com** |
| Sumiya Akhter | **Noakhali Science and Technology University** | **sumaiyahaque338@gmail.com** |
| Md. Maruf Chowdhury | **Rajshahi University** | **marufchowdhury187@gmail.com** |
| Shakila Akter Shilpy | **Rajshahi University** | **shakilashilpi23@gmail.com** |
| Safa Akter Ruma | **Jahangirnagar University** | **safa.ruma101@gmail.com** |
| Samsia Alom Propa | **Rajshahi university** | **samciaalompropa@gmail.com** |
| Mohosina Akther | **Jahangirnagar University** | **mohonakn@gmail.com** |
| Md.Timu Rahman | **Joypurhat Govt college** | **timudewan80@gmail.com** |
| Md Islamul Alam | **University of Dhaka** | **everpranto@gmail.com** |
| Shariful Alam | **Sylhet Engineering college** | **2ashariful@gmail.com** |
| Md Sagor Ali | **Rajshahi University** | **foredurrezasagor@gmail.com** |
| Mt.Shirajum Monira | **Jagannath University** | **shirajumminora.jnu@gmail.com** |
| Shahrin Akter Mim | **Jahangirnagar University** | **shahrin@phiju.edu.bd** |
| Afroza Akter Ankhi | **University of Rajshahi** | **afrozaankhi18@gmail.com** |
| Asfia Shams Chowdhury | **Ibn Sina Medical College** | **shams.pearly97@gmail.com** |
| Md. Abul Hasan | **Hamdard University Bangladesh** | **hasanchowdhury591@gmail.com** |
| Mitu Mony | **Jahangirnagar University** | **rosemitu07@gmail.com** |
| Supti Saha | **University of Chittagong** | **suptisaha358@gmail.com** |
| Atia Munni | **Jahangirnagar University** | **munniphiju46@gmail.com** |
| Mst Nishat Arni | **Rajshahi University** | **ornenishat@gmail.com** |
| Most: Umma Sumaya | **Rajshahi University** | **ummasumaya94@gmail.com** |
| Md. Ashiqur Rahman | **Rajshahi University** | **ashiq.rahman1950@gmail.com** |
| Mst Sumaiya Khatun | **Rajshahi University** | **mstsumaiya14032001@gmail.com** |
| Quazi Asif Ahmed | **Ahsanullah University of Science and Technology** | **ahmedasif.bcic@gmail.com** |
| Rehnuma Rahman | **United International University** | **raisarehnuma1234@gmail.com** |
| Md.Jannatul Nayin | **Noakhali science and Technology University** | **nurmuhammadnayim121@gmail.com** |
| Shahan Ara Taskin Tanha | **Enam Medical College & Hospital** | **taskintanha63@gmail.com** |
| Md Tarikul Islam | **Rajshahi University** | **realtarikulislam@gmail.com** |
| Md.Bakhtiar Abid Chowdhury | **Rajshahi University** | **mohammadbakhtiarabid@gmail.com** |
| Taslima | **Hamdard University Bangladesh** | **taslimajahan988@gmail.com** |
| Faria Sultana Shinjan | **Hamdard University Bangladesh** | **fsshinjan@gmail.com** |
| Mst.Nahid Akter | **Hamdard University Bangladesh** | **mstnahidaktar034@gmail.com** |
| Tanjeem Hasan Prangan | **Rajshahi University** | **tanzimprangan1c@gmail.com** |
| Nayla Jahan Esha | **Hamdard University Bangladesh** | **naylajahanesha@gmail.com** |
| Md. Shariful Islam | **Hamdard University Bangladesh** | **xharif3344@gmail.com** |
| Sabeha Sanjana | **Jagannath University** | **sabehas17@gmail.com** |
| Md Hasibuzzaman Sourov | **University of Chittagong** | **2015sourov@gmail.com** |
| Mst.Sharmin Hoque Mondal | **Hamdard University Bangladesh** | **sharmin934160011@gmail.com** |
| Morsheda Alam Mili | **Jahangirnagar University** | **morsheda@phiju.edu.bd** |
| Md. Ahsan Habib | **Hamdard University Bangladeah** | **www.ahsan.98@gmail.com** |
| Marjan Akter | **Hamdard University Bangladesh** | **mahiamarjaan@gmail.com** |
| Himadri Ranjan Sarkar | **Hamdard University Bangladesh** | **himadrisarkar2016@gmail.com** |
| Mahmuda Akter | **Rajshahi University** | **mitumahmuda962@gmail.com** |
| Sanchita Mondal Tumpa | **University of Dhaka** | **tumpa.idf@gmail.com** |
| Soniya Akter Sony | **Jahangirnagar University** | **soniyaaktersony1999@gmail.com** |
| Tamanna Omar Shapna | **Jagangirnagar University** | **tamanna1473@gmail.com** |
| Rubaiya Binthe Hashem | **Jahangirnagar University** | **rubaiya.gracy11@gmail.com** |
| Mohtasim Monim | **Jahangirnagar University** | **monimju11@gmail.com** |
| Md. Fahad Shahariar Nayon | **Jahangirnagar University** | **fahadshahariar1997@gmail.com** |
| Fahia Rahman | **Rajshahi University** | **fahiarahman06@gmail.com** |
| Abdullah Hasan | **Shahjalal University of Science and Technology** | **hasananp559@gmail.com** |
| Md. Sydul Islam | **Rajshahi University** | **sydulislam1191@gmail.com** |
| Md. Mehedi Hasan | **Rajshahi University** | **mehedihasan4008@gmail.com** |
| Gopal Pal | **Rajshahi University** | **gopalru212@gmail.com** |
| Shahjalal Ahmed Akash | **Jahangirnagar university** | **shahjalalahmed692@gmail.com** |
| Kamrun Nahar Smrity | **Jahangirnagar University** | **kamrunsmrity8751@gmail.com** |
| Mahmuda Akter | **Jahangirnagar University** | **metumahmuda29@gmail.com** |
| Lazin Haque | **Rajshahi University** | **3800lazinhaque@gmail.com** |
| Md. Mahfujur Rahman | **Jagannath University** | **mdmahfujrahman1620@gmail.com** |
| Jannatul Ferdaus | **Hamdard University Bangladesh** | **dr.jannatheaven@gmail.com** |
| Raitah Islam | **Rajshahi University** | **raitahislam7@gmail.com** |
| Israt Jahan Bushra | **Patuakhali science and technology university** | **isratjahanbushra223@gmail.com** |
| Mst.Sanchita Akter | **Hajee Mohammad Danesh science and Technology University** | **sanchitaakter30@gmail.com** |
